# 1α,25-Dihydroxyvitamin D3 Ameliorates Seawater Aspiration-Induced Lung Injury By Inhibiting The Translocation Of NF-κB and RhoA

**DOI:** 10.1007/s10753-017-0527-3

**Published:** 2017-02-04

**Authors:** Minlong Zhang, Faguang Jin

**Affiliations:** Department of Respiration, Tangdu Hospital, Fourth Military Medical University, Xi’an, 710038 People’s Republic of China

**Keywords:** NF-κB, RhoA, acute lung injury, seawater, 1α,25-Dihydroxyvitamin D3

## Abstract

Our previous study have reported that 1α,25-Dihydroxyvitamin D3 (calcitriol) suppresses seawater aspiration-induced ALI *in vitro* and *in vivo*. We also have confirmed that treatment with calcitriol ameliorates seawater aspiration-induced inflammation and pulmonary edema *via* the inhibition of NF-κB and RhoA/Rho kinase pathway activation. In our further work, we investigated the effect of calcitriol on nuclear translocation of NF-κB and membrane translocation of RhoA *in vitro*. A549 cells and rat pulmonary microvascular endothelial cells (RPMVECs) were cultured with calcitriol or not for 48 h and then stimulated with 25% seawater for 40 min. After these treatments, cells were collected and performed with immunofluorescent staining to observe the translocation of NF-κB and RhoA and the cytoskeleton remodeling. *In vitro*, seawater stimulation activates nuclear translocation of NF-κB and membrane translocation of RhoA in A549 cells. In addition, seawater administration also induced cytoskeleton remodeling in A549 cells and RPMVECs. However, pretreatment with calcitriol significantly inhibited the activation of NF-κB and RhoA/Rho kinase pathways, as demonstrated by the reduced nuclear translocation of NF-κB and membrane translocation of RhoA in A549 cells. Meanwhile, treatment of calcitriol also regulated the cytoskeleton remodeling in both A549 cells and RPMVECs. These results demonstrated that treatment with calcitriol ameliorates seawater aspiration-induced ALI *via* inhibition of nuclear translocation of NF-κB and membrane translocation of RhoA and protection of alveolar epithelial and pulmonary microvascular endothelial barrier.

## INTRODUCTION

Drowning is a major cause of unintentional injury death [1]. Acute lung injury (ALI) or acute respiratory distress syndrome (ARDS) is a serious body injury induced by seawater aspiration [2]. Seawater aspiration-induced ALI is characterized by the inflammatory process in pulmonary parenchyma and interstitial tissue and severe pulmonary edema [3]. It has been reported that increased alveolar epithelial and pulmonary microvascular endothelial permeability triggered the transmigration of inflammatory cells such as neutrophils and the formation of edema fluid in alveolar. Earlier work from our laboratory also found that seawater administration induced the transmigration of inflammatory cells and increased pulmonary epithelial-endothelial barrier permeability [4].

Nuclear factor kappa B (NF-κB) signaling pathway is an important pathway in inflammatory responses [5–7]. Several pro-inflammatory stimuli can cause the activation of NF-κB through the phosphorylation of inhibitors of κB (IκBs) by the IκB kinase (IKK) complex [8]. Afterwards, the phosphorylated NF-κB translocate into the nucleus in which it can lead to the transcriptional activation of several pro-inflammatory mediators. Rho and its target protein, Rho-associated coiled-coil forming protein kinase(ROCK) pathways implicated in the cytoskeletal contractile response through their influence on myosin ATPase activity [9, 10]. It has been reported that Rho and ROCK are well-established mediators of the permeability between cells. In our previous study, seawater stimulation also activated the NF-κB and RhoA/Rho kinase pathways [11].

1α,25-Dihydroxyvitamin D3 (calcitriol) is the active form of vitamin D. several studies have reported that calcitriol can inhibit neutrophil recruitment and pro-inflammatory cytokines release and reduce the actin-dependent cytoskeletal rearrangement through RhoA/ROCK pathway [12, 13]. Our previous study found that calcitriol improved lung histopathologic changes, reduced inflammation, lung edema, and vascular leakage in seawater-induced ALI. In addition, calcitriol also significantly inhibited the activation of NF-κB and RhoA/Rho kinase pathways.

On the basis of our previous study [4], we investigated the effect of calcitriol on nuclear translocation of NF-κB and membrane translocation of RhoA in seawater stimulated alveolar epithelial cells. Besides, we also observed the effect of calcitriol on cytoskeleton remodeling in both epithelial and endothelial cells. Dexamethasone treatment has been proved to have therapeutic effects in seawater aspiration-induced ALI. Therefore, we choose dexamethasone as a positive control drug.

## MATERIALS AND METHODS

### Animal Preparation

Sprague-Dawley (SD) rats (male, 5–7 weeks old, 200 ± 20 g) were obtained from the Animal Center of Fourth Military Medical University. The feeding environment of rats includes temperature-controlled house with 12-h light-dark cycles, free access to standard laboratory diet and water *ad libitum*. All the animal experiments were approved by the Animal Care and Use Committee of the Fourth Military Medical University and in accordance with the Declaration of the National Institutes of Health Guide for Care and Use of Laboratory Animals (Publication No.85-23, revised 1985).

### Drug and Reagents

Seawater (osmolality 1300 mmol/L, PH 8.2, SW 1.05, NaCl 6.518 g/L, MgSO_4_ 3.305 g/L, MgCl_2_ 2.447 g/L, CaCl_2_ 1.141 g/L, KCl 0.725 g/L, NaHCO_3_0.202 g/L, NaBr 0.083 g/L) was prepared according to the major composition of the East China Sea provided by Chinese Ocean Bureau. Anti-RhoA and anti-CD31 antibodies were purchased from Santa Cruz Biotechnology Inc. (Santa Cruz, CA, USA). Anti- NF-κB p65 antibodies were purchased from Cell Signaling Technology (Cell Signaling, MA, USA). Anti-Pan-Cadherin antibodies were purchased from Epitomics (Burlingame, CA, USA).

### A549 Cell Culture and Treatment

A human lung epithelial cell line, A549 (obtained from ATCC, Rockville, MD, USA), was maintained in RPMI 1640 medium supplemented with 100 U/ml of penicillin-streptomycin and 10% fetal bovine serum (FBS) at 37 °C in a humidified atmosphere containing 5% CO_2_ and 95% air. After incubated in the presence or absence of calcitriol (10^−6^M) and dexamethasone (10^−6^M) 48 h, seawater (0.25 ml per 1 ml total volume) were added to A549 cells and the cells were stimulated for indicated time.

### RPMVEC Isolation, Treatment, and Identification

Primary RPMVECs isolation and culture were performed according to previous methods with some modification [14]. Firstly, the pleura and outer edges of washed fresh rat lung lobe were cut off. Then, the 1.5-mm^3^ specimens of tissue cut from lung surface were carefully plated into cell culture dishes (containing DMEM supplemented with 20% FBS, 25 μg/ml of endothelial cell growth supplement and 100 U/ml of penicillin-streptomycin). These tissues were cultured at 37 °C in a humidified atmosphere with 5% CO_2_ and 95% air. The residue specimens were removed after 60 h. The cells were passed (with 0.25% trypsin) when monolayer cells were achieved and all experimental cells were between passages 2 and 3. Primary RPMVECs were identified according to their characteristic morphology and staining with anti-CD31 antibody. After incubated in the presence or absence of calcitriol (10^−6^M) and dexamethasone (10^−6^M) 48 h, seawater (0.25 ml per 1 ml total volume) were added to cells and the cells were stimulated for indicated time.

### Immunofluorescent Staining

For NF-κB translocation staining, cells were seeded in growth medium onto sterile glass slides and incubated for 24 h with calcitriol (10^−6^M) and dexamethasone (10^−6^M), then challenged with 25% seawater for 40 min. Cells in serum-free growth medium were used as controls. Cells were then fixed with 4% paraformaldehyde (pH7.4) for 10 min, permeabilized for 10 min with PBS containing 0.1% TritonX-100, and incubated with 2% bovine serum albumin (BSA) for 30 min. Immunostaining was performed using rabbit NF-κB p65 antibody (1:100 dilution), followed by polyclonal anti-rabbit IgG FITC (1:200 dilution, Bioss, Beijing, China). After washing, cells were stained with 5 μg/ml 4′, 6-diamidino-2-phenylindole dihydrochloride (DAPI, Sigma-Aldrich) for 10 min at room temperature. Slides were then washed and mounted with ProLong Gold anti-fade reagent (Invitrogen) and read with fluorescence microscope (Olympus) at ×40. Negative controls were performed by incubation with appropriate isotype-matched primary Abs.

For RhoA staining, cells were seeded in growth medium onto sterile glass slides and incubated for 24 h with calcitriol (10^−6^M) and dexamethasone (10^−6^M), then challenged with 25% seawater for 40 min. Cells in serum-free growth medium were used as controls. Cells were then fixed with 4% paraformaldehyde (pH 7.4) for 10 min, permeabilized for 10 min with PBS containing 0.1% TritonX-100, and incubated with 2% bovine serum albumin (BSA) for 30 min. Immunostaining was performed using rabbit RhoA antibody (1:100 dilution) and mouse pan-cadherin antibody (1:100 dilution), followed by rhodamine red goat anti-mouse IgG (1:200 dilution, Bioss, Beijing, China) and anti-rabbit IgG FITC (1:200 dilution). After washing, cells were stained with 5 μg/ml DAPI for 10 min at room temperature. Slides were then washed and mounted with ProLong Gold anti-fade reagent and read with confocal microscope (Olympus) at ×60.

The F-actin was stained with fluorochrome-conjugated phalloidin (Alexa488-Phalloidin; Molecular Probes, Eugene, OR) for 60 min at room temperature. Nuclei were stained with DAPI. Slides were mounted with ProLong Gold anti-fade reagent and read with confocal microscope at ×60.

## RESULTS

### Effects of Calcitriol on NF-κB p65 Nuclear Translocation in A549 Cells after Seawater Treatment

**Fig. 1:**
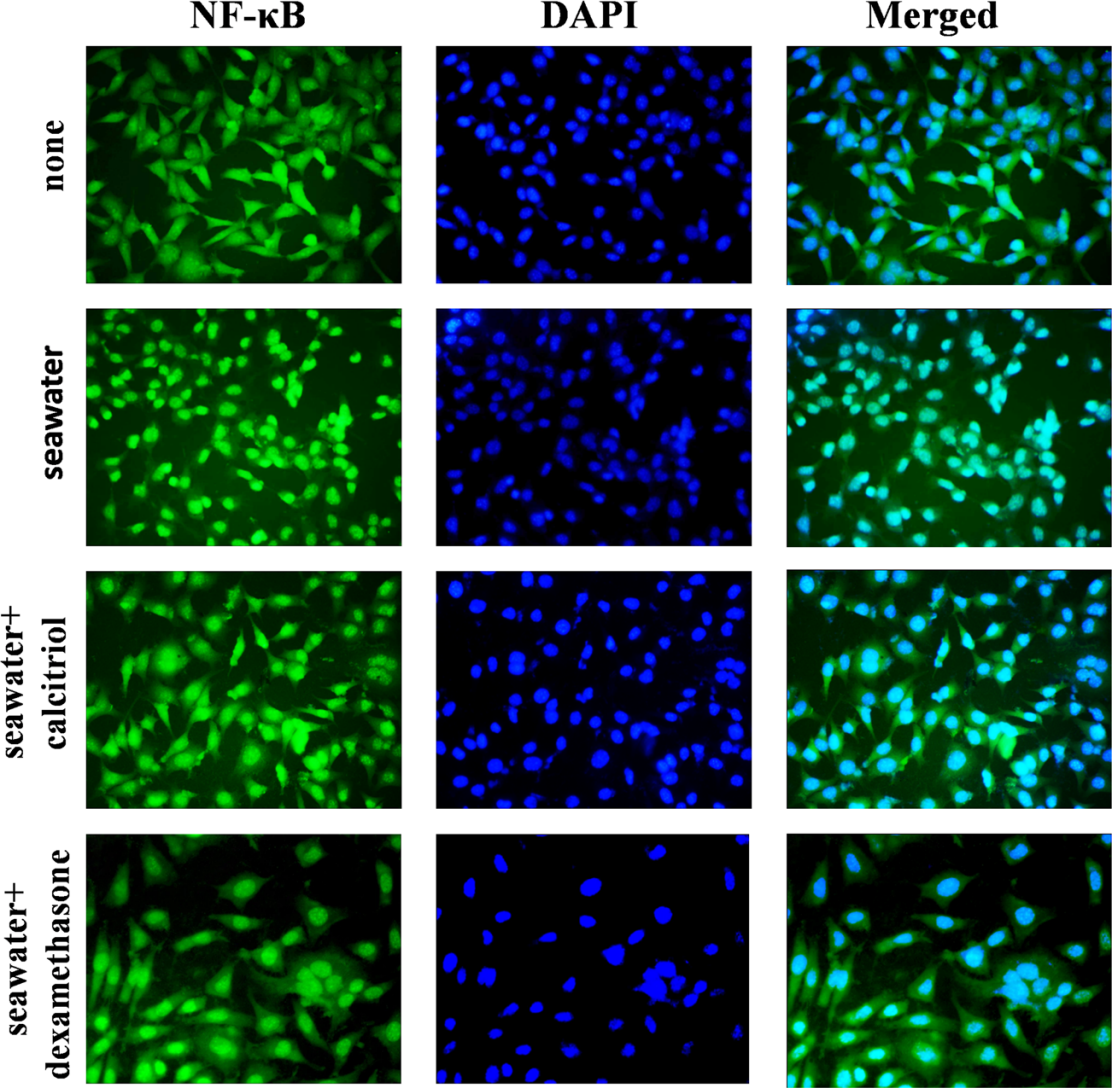
Effects of calcitriol on nuclear translocation in A549 cells. After treatment, cells were washed, fixed, and stained with anti- NF-κB p65 antibody and DAPI. Cells were analyzed by fluorescence microscope, and individual and merged stainings are shown (magnification ×40). NG: normal group; SG: seawater group; CG: 10^−6^M calcitriol group; DG: dexamethasone group.

To identify the effect of calcitriol to inhibit translocation of NF-κB p65 to the nucleus in A549 cells, cells were cultured for 24 h with or without calcitriol followed by stimulation for 40 min with seawater. Individual and merged stainings were obtained by fluorescence microscopic analysis (Fig. 1). As shown in the figure, NF-κB p65 translocated into the nucleus in seawater-stimulated A549 cells. However, NF-κB p65 was retained into the cytoplasm in A549 cells treated with calcitriol. Therefore, calcitriol inhibited the nuclear translocation of NF-κB p65. Moreover, similar effect was observed in dexamethasone group.

### Effects of Calcitriol on RhoA Plasma Membrane Translocation in A549 Cells Stimulated by Seawater

**Fig. 2:**
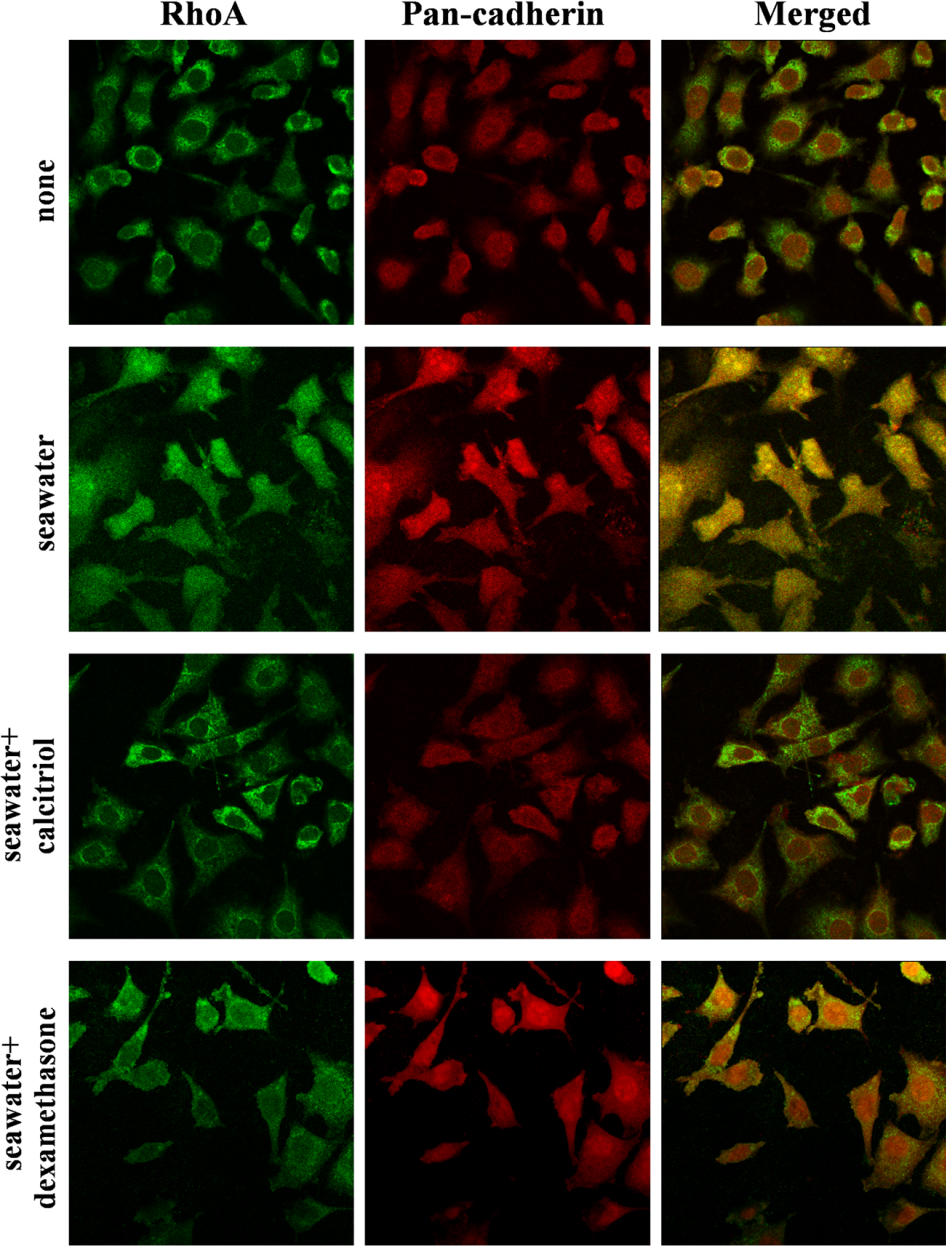
Effects of calcitriol on RhoA translocation in A549 cells. After treatment, cells were washed, fixed, and stained with anti-RhoA and anti-pancadherin antibodies. Cells were analyzed by confocal microscopy, and individual and merged stainings are shown (magnification ×60). NG: normal group; SG: seawater group; CG: 10^−6^M calcitriol group; DG: dexamethasone group.

As RhoA activation results in its translocation to the cell plasma membrane, we used immunofluorescence to validate these results. The intracellular localization of RhoA was monitored by staining with specific anti-RhoA antibody and compared with pan-cadherin immunoreactivity as a cell membrane marker (Fig. 2). This figure confirmed that seawater stimulation increased RhoA localization at the plasma membrane. Whereas, RhoA was retained in the cytoplasm in A549 cells treated with calcitriol and similar effect was observed in dexamethasone group. Therefore, calcitriol inhibited the membrane translocation of RhoA.

### Effects of Calcitriol on Cytoskeletal Remodeling in A549 Cells Stimulated by Seawater

**Fig. 3:**
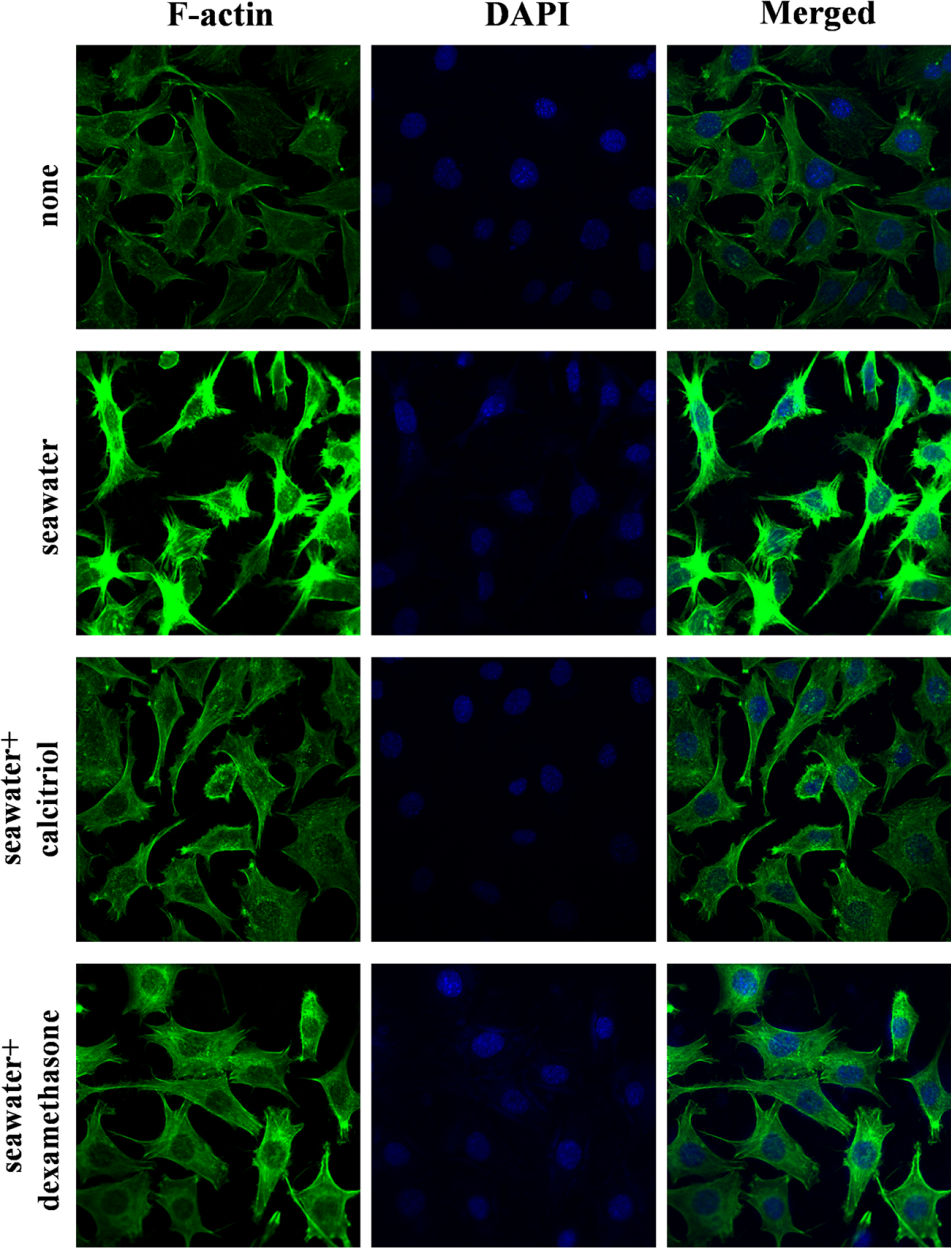
Effects of calcitriol on cytoskeletal remodeling in A549 cells. A549 cells were treated for 40 min with seawater in the presence or absence of calcitriol and dexamethasone. Phenotype of cultured A549 cells were examined by confocal microscopy following staining for F-actin (magnification ×60). NG: normal group; SG: seawater group; CG: 10^−6^M calcitriol group; DG: dexamethasone group.

Cell contraction is a RhoA/ROCK-dependent event. To evaluate the effect of calcitriol on cytoskeletal remodeling as a parameter of pulmonary barrier function, we stained F-actin on A549 cells (Fig. 3). Compared with control cells, seawater treatment induced an evident motile phenotype characterized by increase of stress fibers, filopodia and membrane ruffles. Pretreatment with calcitriol and dexamethasone led to significantly reduction of the actin-dependent cytoskeletal remodeling.

### Effects of Calcitriol on Cytoskeletal Remodeling in RPMVECs Stimulated by Seawater

**Fig. 4:**
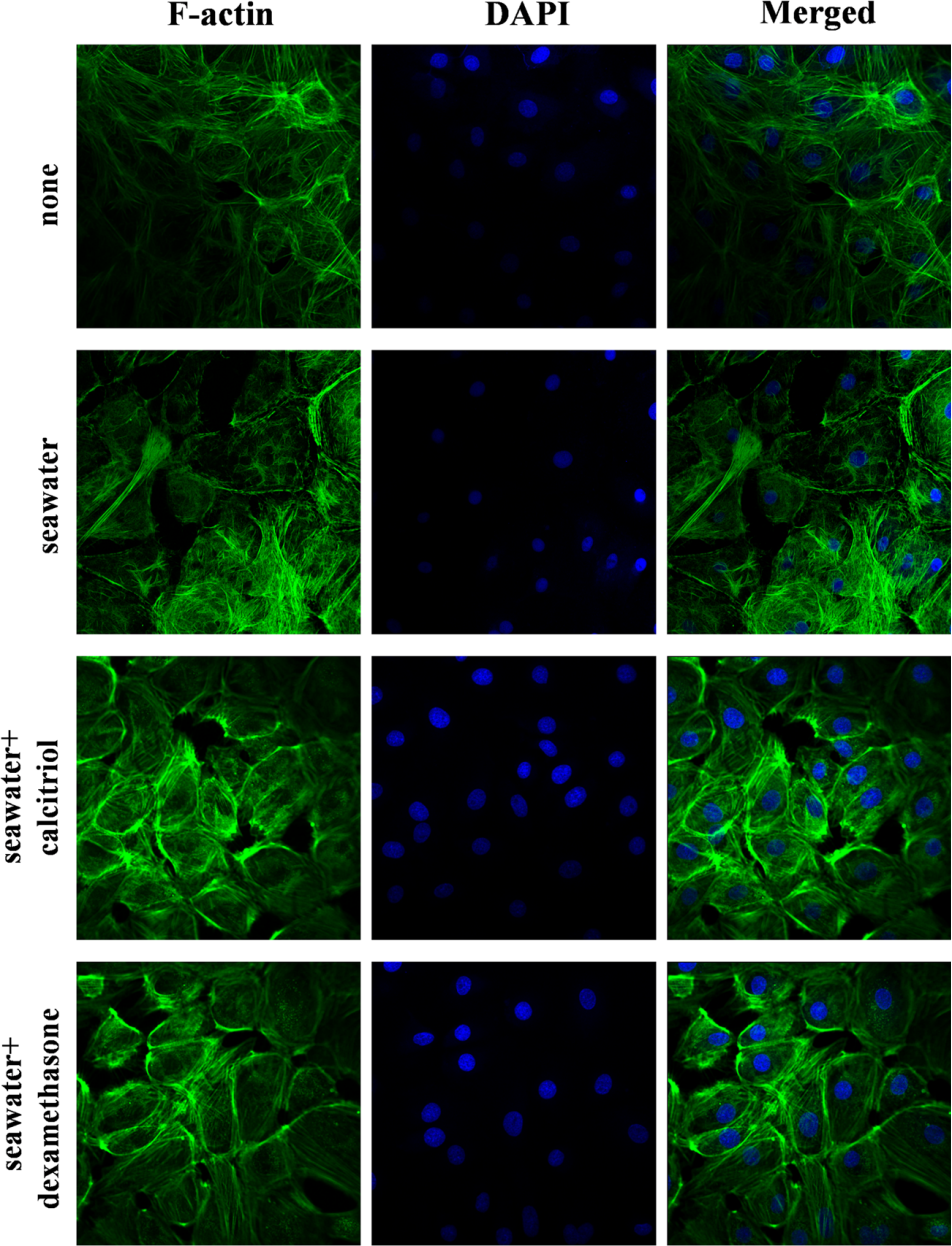
Effects of calcitriol on cytoskeletal remodeling in RPMVECs. RPMVECs were treated for 40 min with seawater in the presence or absence of calcitriol and dexamethasone. Phenotype of cultured RPMVECs were examined by confocal microscopy following staining for F-actin (magnification ×60). NG: normal group; SG: seawater group; CG: 10^−6^M calcitriol group; DG: dexamethasone group.

To assess the effect of calcitriol on cytoskeletal remodeling in RPMVECs, we stained F-actin on cells (Fig. 4). As shown in the figure, seawater exposure induced the increase of stress fibers in RPMVECs, but the effect was less prominent than in A549 cells. Furthermore, calcitriol and dexamethasone reduced the actin-dependent cytoskeletal remodeling. Nevertheless, no significant difference between calcitriol group and dexamethasone group was observed.

## DISCUSSION

In our present study, we investigated the effect of calcitriol on nuclear translocation of NF-κB and membrane translocation of RhoA *in vitro*. In addition, we explored the effect of calcitriol on cytoskeletal remodeling in lung epithelial-endothelial barrier. These results showed that seawater stimulation activates nuclear translocation of NF-κB and membrane translocation of RhoA in A549 cells. Seawater administration also led to cytoskeletal remodeling both in A549 cells and RPMVECs. However, calcitriol significantly inhibited nuclear translocation of NF-κB and membrane translocation of RhoA. Moreover, calcitriol also regulated the cytoskeleton remodeling in both A549 cells and RPMVECs.

Inflammation and lung epithelial-endothelial barrier injury are two main characteristics in seawater aspiration-induced ALI. NF-κB pathway activation plays an important part in seawater aspiration-induced ALI. Seawater simulation may lead NF-κB phosphorylation, and phosphorylated NF-κB translocation from the cytoplasm to the nucleus is a key step in the expression of downstream inflammation-related genes [15]. RhoA/Rho kinase pathway is critical in the cytoskeletal contractile response. Several published works suggested that RhoA/ROCK pathway can also activate NF-κB and these findings indicated that RhoA/ROCK pathway is a potential pathway in inflammation responses [16]. RhoA belongs to monomeric GTP-binding proteins and GTP-RhoA cytoplasm membrane translocation is a critical step in the inflammation process and cytoskeletal contractile response [17]. As is known to all, increased lung tissue barrier permeability can enhance inflammatory cells recruitment [18]. Thus, translocation of activated NF-κB and RhoA may have important roles in the process of seawater aspiration-induced ALI. Our previous study has confirmed that seawater stimulation resulted in NF-κB and RhoA/ROCK pathways activation. In present work, we further demonstrated that seawater administration stimulated NF-κB nuclear translocation and RhoA membrane translocation. In addition, seawater also promoted the cytoskeletal remodeling both in epithelial and endothelial cells.

Several works have confirmed that, active metabolite of vitamin D, calcitriol can suppress inflammatory responses and inhibit neutrophil infiltration [19, 20]. Previous study in our lab also demonstrated that calcitriol ameliorated seawater aspiration-induced inflammation and pulmonary edema and these effects worked through inhibition of NF-κB and RhoA/ROCK pathways activation. In present work, we further confirmed that calcitriol inhibited nuclear translocation of NF-κB and membrane translocation of RhoA. Furthermore, calcitriol also regulated the cytoskeleton remodeling in both epithelial and endothelial cells.

In summary, our work demonstrated that pre-treatment with calcitrol ameliorates seawater aspiration-induced ALI *via* inhibition of nuclear translocation of NF-κB and membrane translocation of RhoA and protection of alveolar epithelial and pulmonary microvascular endothelial barrier.
